# Supplementary figures and images for: High Degree of Polymerization of Chitin Oligosaccharides Produced from Shrimp Shell Waste by Enrichment Microbiota Using Two-Stage Temperature-Controlled Technique of Inducing Enzyme Production and Metagenomic Analysis of Microbiota Succession
Source: Mar Drugs. 2024 Jul 28;22(8):346. doi: 10.3390/md22080346 (PMC11355434; doi:10.3390/md22080346)

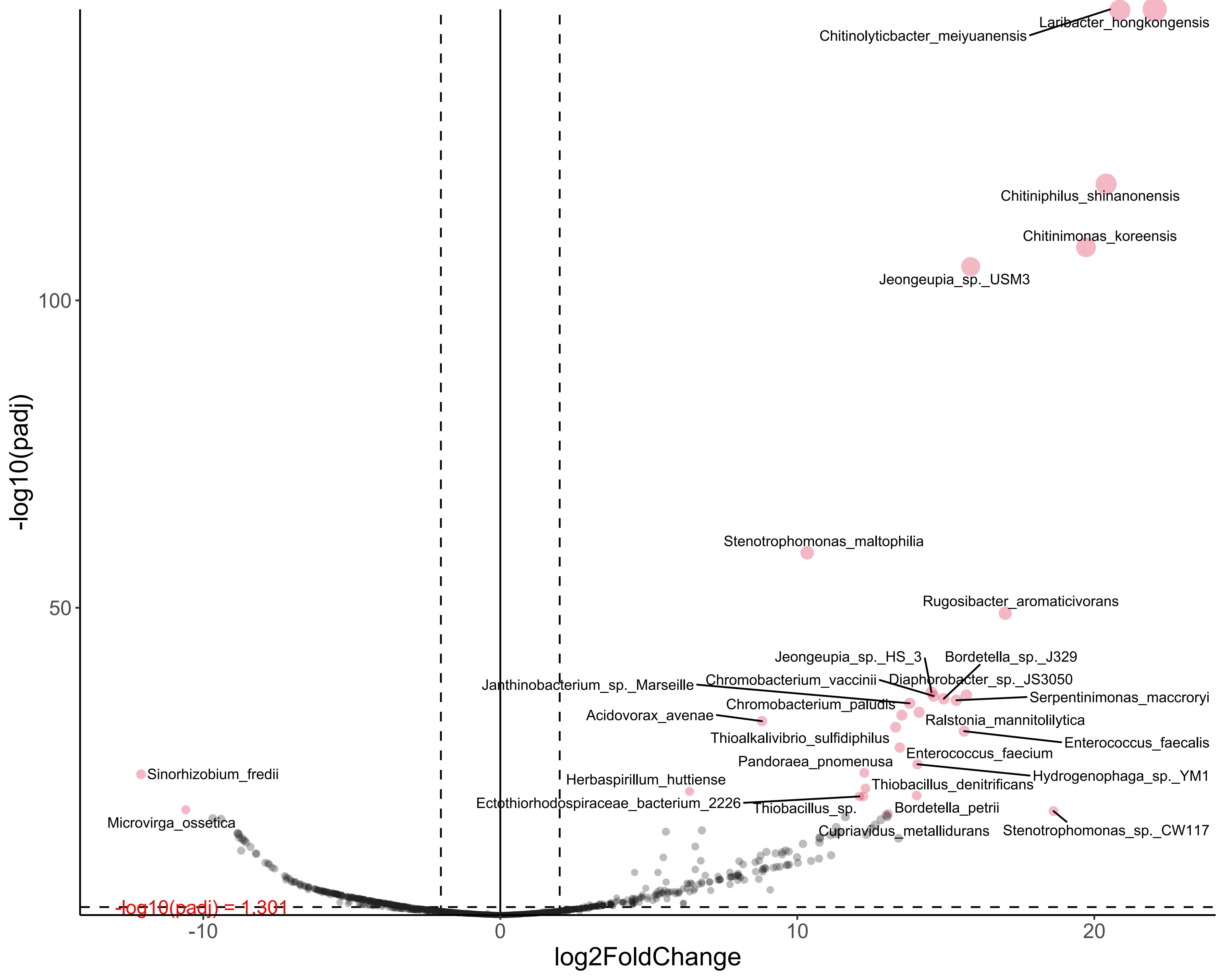

Supplement: Supplementary file 1 [file marinedrugs-22-00346-s001.zip › Supplementary Materials/Figure S1.jpg]

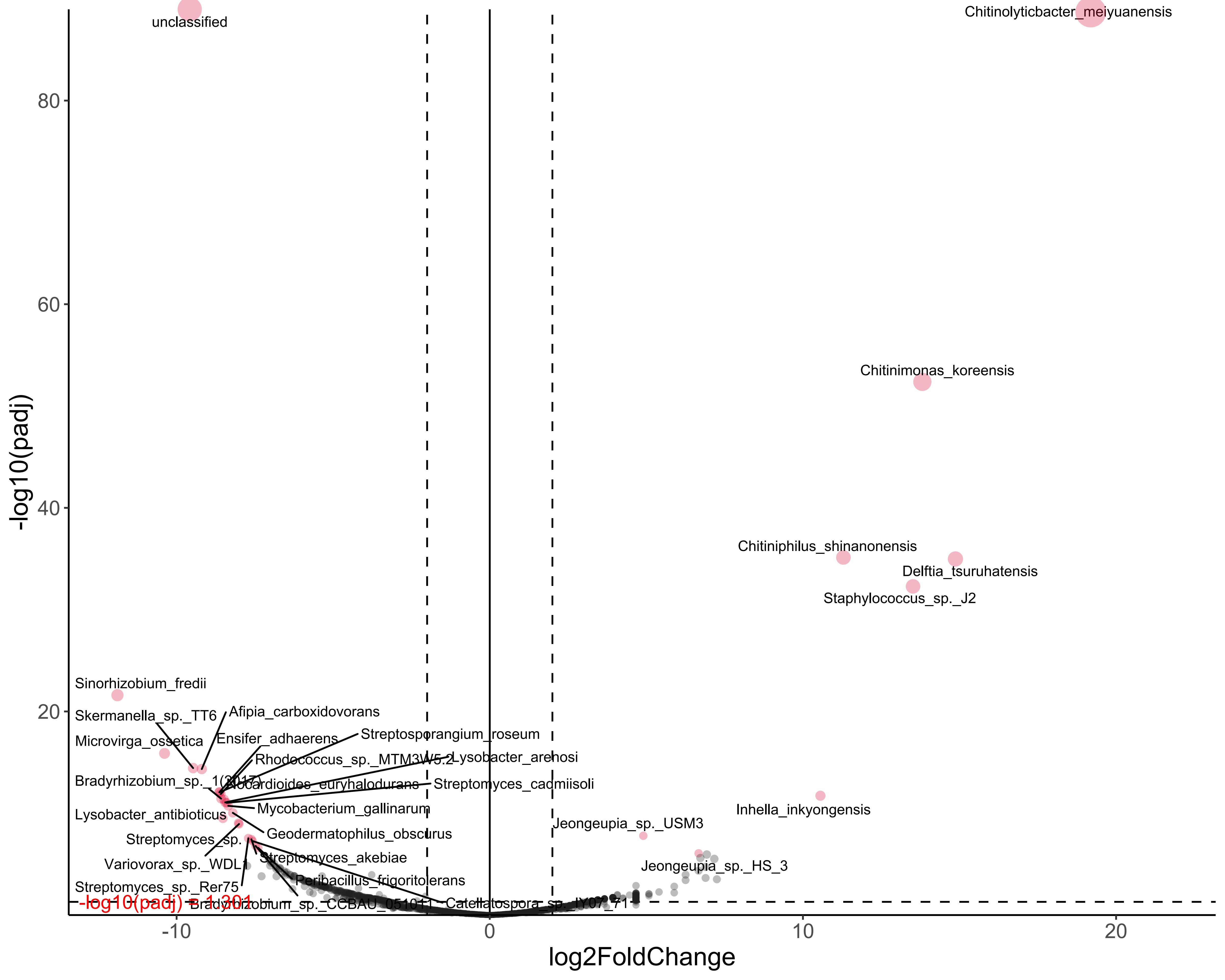

Supplement: Supplementary file 1 [file marinedrugs-22-00346-s001.zip › Supplementary Materials/Figure S2.jpg]
